# Efficacy and Safety of Andexanet Alfa for Bleeding Caused by Factor Xa Inhibitors: A Systematic Review and Meta-Analysis

**DOI:** 10.7759/cureus.20632

**Published:** 2021-12-23

**Authors:** Dhan B Shrestha, Pravash Budhathoki, Ayush Adhikari, Sudat Shrestha, Nirajan Khati, Wasey Ali Yadullahi Mir, Tilak Joshi, Anuj Shrestha

**Affiliations:** 1 Department of Internal Medicine, Mount Sinai Hospital, Chicago, USA; 2 Department of Internal Medicine, Bronxcare Health System, Bronx, USA; 3 Department of Internal Medicine, Tribhuvan University Institute of Medicine, Kathmandu, NPL; 4 Department of Surgical Intensive Care Unit, Patan Academy of Health Sciences, Lalitpur, NPL; 5 Department of Emergency Medicine, Bishnudevi Hospita, Kirtipur, NPL; 6 Department of Internal Medicine, Mount Sinai Medical Center, Chicago, USA; 7 Department of Medicine, Section of Hematology/Oncology, University of Missouri–Kansas City School of Medicine, Kansas City, USA

**Keywords:** cerebral hemorrhage, meta-analysis, bleeding reversal, factor xa inhibitors, andexanet alfa

## Abstract

Direct oral anticoagulants (DOAC) including factor Xa inhibitors are associated with bleeding events which can lead to severe morbidity and mortality. Reversal agents like andexanet alfa (AA) and 4F-PCC (Four-factor prothrombin concentrate complex) are available for treating bleeding that occurs with DOAC therapy but a comparison on their efficacy is lacking. In this study, we analyzed the efficacy and safety of patients treated with andexanet alfa for bleeding events from DOAC. Databases were searched for relevant studies where AA was used to determine efficacy and safety in bleeding patients who were on factor Xa inhibitors. Published papers were screened independently by two authors. RevMan 5.4 (The Cochrane Collaboration, 2020) was used for data synthesis. Odds ratio (OR) and mean difference (MD) was used to estimate the outcome with a 95% confidence interval (CI). Among 1245 studies were identified after a thorough database search and three studies were included for analysis. AA resulted in lower odds of mortality compared to 4F- PCC (OR, 0.37; 95% CI, 0.20-0.71) among patients with intracerebral hemorrhage. There was no difference in thrombotic events between patients receiving AA and 4F-PCC (OR, 2.40; 95% CI, 0.36-15.84). No differences in length of hospital stay and intensive care unit (ICU) stay were seen between patients receiving AA and 4F-PCC. In conclusion, andexanet alfa reduced in-hospital mortality in patients who had bleeding due to factor Xa inhibitors compared to 4F-PCC. However, there were no differences in thrombotic events, length of ICU, and hospital stay between patients treated with AA and 4F-PCC.

## Introduction and background

Direct oral anticoagulants (DOAC) have been increasingly used in patients for the prevention of systemic embolization in atrial fibrillation as well as treatment and prevention of deep vein thrombosis (DVT) and venous thromboembolism (VTE). As a result, the indications of DOAC have significantly expanded in the last decade [1-5]. Predictable pharmacokinetics and pharmacodynamics, rapid onset and offset of action, few drug interactions, and absence of need for regular laboratory monitoring provide an advantage to oral factor Xa inhibitors over traditional Vitamin K antagonists [6]. Factor Xa inhibitors also reduce fatal and intracranial hemorrhage compared with vitamin K antagonists [7,8]. However, fatal bleeding has been reported with oral factor Xa inhibitor use [8,9].

Before the introduction of andexanet alfa (AA), off-label use of 4 factor-prothrombin concentrate complex (4F-PCC) was advised and was used in the situation of life-threatening bleeding [10]. Prothrombin complex concentrates (PCCs) are isolated from fresh frozen plasma (FFP) and contain Vitamin K-dependent factors II, VII, IX, and X [11]. In May 2018, AA received FDA approval for use in patients treated with rivaroxaban and apixaban in the setting of life-threatening or uncontrolled bleeding following ANNEXA-A and ANNEXA-R trials in healthy participants [12,13]. AA is a modified recombinant, catalytically inactive form of human factor Xa, which binds and sequesters factor Xa inhibitor molecules that reduce anti-factor Xa activity rapidly in the body [14]. A multicenter, prospective, open-label, single-group study ANNEXA-4 was done in bleeding patients following FDA approval, which showed the drug's good efficacy and safety profile [15]. Randomized controlled trials have not been done, given the risks of using a placebo in acutely bleeding patients. However, some retrospective observational studies and case series studying the efficacy and safety of AA in bleeding patients have been published. In addition, some studies have compared efficacy and safety with 4F-PCC. We have conducted this systematic review and meta-analysis to analyze the effectiveness and safety profile of AA in bleeding caused by factor Xa inhibitors.

## Review

Methods

We used Preferred Reporting Items for Systematic Reviews and Meta-Analysis (PRISMA) guidelines for the systematic review of available literature [16]. The study protocol was registered in the International prospective register of systematic reviews (PROSPERO) CRD42021244219.

Literature search

We searched PubMed, PubMed Central, Scopus, Embase, and Cochrane library for relevant studies published till February 2021. Searches were conducted using the keywords like "andexanet alfa", "andexanet", "andexanet alpha", "bleeding", "factor Xa inhibitor," and "factor Xa inhibitors" and appropriate boolean operators. Details of the search strategy are available in Supplementary Material 1.

Selection of studies

A. Types of Studies

We included studies done to determine the efficacy and safety of andexanet alfa in patients who had bleeding in the setting of factor Xa inhibitor use. As randomized controlled trials were not available, we included prospective and retrospective studies and case series with more than ten patients. AA was used to determine efficacy and safety in bleeding patients on factor Xa inhibitors in qualitative analysis. In addition, the studies with both treatment and control groups were included in the quantitative synthesis.

B. Types of Participants

The studies required patients to be more than 18 years of age and had bleeding in the setting of Factor Xa inhibitor use.

C. Types of Interventions

Andexanet alfa was taken in the treatment arm, while 4F-PCC or other blood products were included in the control arm.

D. Types of Outcome Measures

Our outcome of interest was hemostatic efficacy, mortality within 30 days, the incidence of thrombotic events, and length of hospital and ICU stay following treatment with AA or other blood products. 

We excluded types of studies with the following characteristics: meta-analysis, reviews, in-vitro studies, studies done on healthy subjects, case reports, editorials, opinions, letters, protocols, abstracts/presentations, dissertation, and animal studies. Case series with fewer than ten patients, articles where full-text articles were not available, ongoing studies, and studies with incomplete data were also excluded.

Data extraction and management

Titles, abstracts, and full texts were screened for study and report characteristics that matched eligibility criteria. Studies were independently screened by two reviewers (AA and SS) using Covidence (Covidence systematic review software, Veritas Health Innovation, Melbourne, Australia) and data were extracted for both quantitative and qualitative synthesis. The conflicts were resolved by taking the opinion of the third reviewer (NK). The data extraction sheet was created using Microsoft Excel software. One reviewer collected the data from all articles; the second reviewer verified the data for accuracy and highlighted discrepancies; the third reviewer resolved any disagreements and carried out a thorough evaluation to ensure that only the outcomes of interest were taken into account. The following variables were included: first author, type of design, site of study, year of publication, sample size, mean age, percentage of male and female, indication for anticoagulation, hemostatic efficacy, mortality within 30 days, length of hospital stay, length of ICU stay and incidence of thrombotic events. 

Risk of Bias

We used the Joanna Briggs Institute (JBI) critical appraisal checklist for cohorts and case series for quality and risk of bias assessment (Tables 1-2).

**Table 1:** JBI Critical Appraisal of Cohort Studies JBI: Joanna Briggs Institute

Questions (Yes, No, Unclear, Not applicable)	Ammar et al. [17]	Barra et al. [18]	Coleman et al. [19]
1. Were the two groups similar and recruited from the same population?	Yes	No	Yes
2. Were the exposures measured similarly to assign people to both exposed and unexposed groups?	Yes	Yes	Unclear
3. Was the exposure measured in a valid and reliable way?	Yes	Yes	Unclear
4. Were confounding factors identified?	Yes	Yes	Yes
5. Were strategies to deal with confounding factors stated?	Yes	Yes	No
6. Were the groups/participants free of the outcome at the start of the study (or at the moment of exposure)?	Yes	Yes	Yes
7. Were the outcomes measured in a valid and reliable way?	Yes	Yes	Yes
8. Was the follow-up time reported and sufficient to be long enough for outcomes to occur?	Yes	Yes	Yes
9. Was follow-up complete, and if not, were the reasons for loss to follow-up described and explored?	Yes	Yes	Yes
10. Were strategies to address incomplete follow-up utilized?	N/A	N/A	N/A
11. Was appropriate statistical analysis used?	Yes	Yes	Yes
Overall Appraisal	Include	Include	Include

**Table 2:** JBI critical appraisal of case series JBI: Joanna Briggs Institute

QUESTION	Brown et al. 2019 [20]	Connolly et al. 2019 [21]	Culbreth et al. 2019 [22]	Culbreth et al. 2018 [23]	Giovino et al. 2020 [24]	Nederpelt et al. 2020 [25]	Stevens et al. 2019 [26]
1) Were there clear criteria for inclusion in the case series?	Yes	Yes	No	Yes	Yes	Yes	Yes
2) Was the condition measured in a standard, reliable way for all participants included in the case series?	Yes	Yes	Yes	Yes	Yes	Yes	Yes
3) Were valid methods used for identification of the condition for all participants included in the case series?	Yes	Yes	Yes	Yes	Yes	Yes	Yes
4) Did the case series have consecutive inclusion of participants?	Yes	No	Yes	Yes	Yes	Yes	Yes
5) Did the case series have the complete inclusion of participants?	Yes	No	Yes	Yes	Yes	Yes	Yes
6) Was there clear reporting of the demographics of the participants in the study?	Yes	Yes	No	No	Yes	Yes	Yes
7) Was there clear reporting of clinical information of the participants?	Yes	Yes	Yes	Yes	Yes	Yes	Yes
8) Were the outcomes or follow-up results of cases reported?	Yes	Yes	Yes	Yes	Yes	Yes	Yes
9) Was there clear reporting of the presenting site(s)/clinic(s) demographic information?	Yes	Yes	No	No	Yes	Yes	Yes
10) Was statistical analysis appropriate?	Yes	Yes	No	No	Yes	Yes	Yes

Statistical Analysis

RevMan 5.4 (The Cochrane Collaboration, 2020) was used for statistical analysis. Odds ratio (OR) and mean difference (MD) was used to estimate the outcome with a 95% confidence interval (CI). 

Assessment of Heterogeneity

The statistical heterogeneity among the studies was calculated and assessed with the I^2^ test based on previously recommended stratifications. In the case of heterogeneity, we used the invariance and random-effect finally, well. Finally, we evaluated the sensitivity by rerunning the analysis to assess any unrevealed differences.

Results

**Figure 1:**
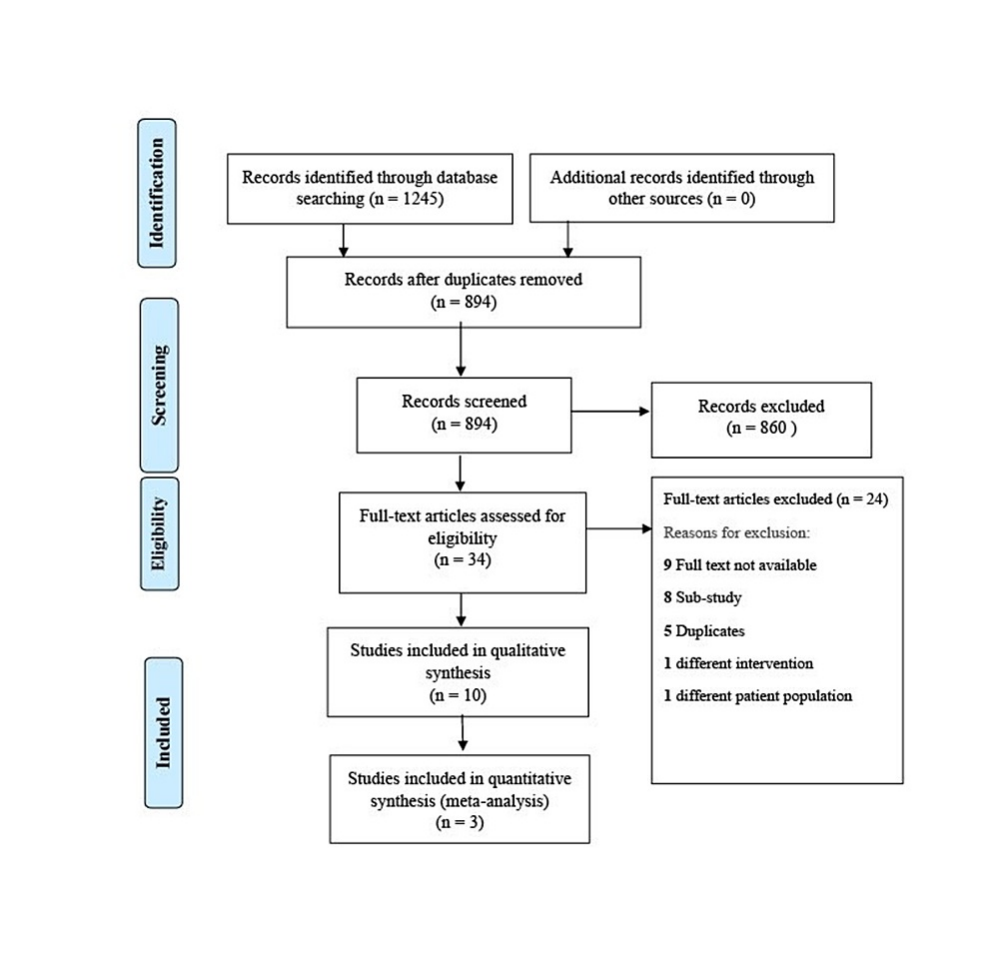
PRISMA Flow Diagram

A total of 1245 studies were identified after thorough database searching, and 351 duplicates were removed. Title and abstracts of 894 studies were screened, and 860 irrelevant studies were excluded. The full-text eligibility of 34 studies was assessed, and 24 studies were excluded for definite reasons (Figure 1). A total of 10 studies were included in the qualitative summary (Table 3), and three studies were included in the quantitative analysis.

**Table 3:** Narrative summary of the included studies FXai: factor Xa inhibitor; GCS: Glasgow Coma Scale; DVT: deep vein thrombosis; ICH: intracerebral hemorrhage; IPH: intraparenchymal hemorrhage; MI: myocardial infarction; TIA: transient ischaemic attack; PE: pulmonary embolism; VTE: venous thromboembolism; 4F-PCC: four-factor prothrombin complex concentrate; N: total number, C: control group, T: treatment group

Study ID	Population	Intervention	Comparator	Outcome
Ammar et al., 2021. A retrospective single-center cohort study, US [17]	Patients with life-threatening traumatic or spontaneous intracranial bleeds in the setting of FXai (apixaban or rivaroxaban) use: N=44 (T=28, C=16); male: T=61%, C=69%; female: T=39%, C=31%; age (median, IQR) T: 78 (70–87), C: 80 (74–84); GCS on admission (median, IQR), T: 14 (11–15), C: 14 (7–15); indication for anticoagulation: T: Afib 21/28, DVT 6/28, other 1/28 C: Afib 13/16, DVT 3/16; FXa inhibitor: T: apixaban 19, rivaroxaban 9, C: apixaban 12, rivaroxaban 4	T: Andexanet alfa low dose or high dose based on product labeling, low dose: 400 mg iv bolus followed by 480 mg infusion. High dose: 800 mg iv bolus followed by 960 mg infusion; low dose 22/28 (79%), high dose 6/28 (21%)	C: 4F-PCC 25 units/kg up to 2500 units per dose	Stable CT scan head at six hours T: 21/28 C: 10/16; stable CT scan head at 24 hours T: 15/28 C: 6/16; IPH baseline hematoma volume T: 8.5 (5.8–23) C: 11 (8.3–46.6); spontaneous IPH hematoma volume at six hours post-reversal T: 9.3 (6.9–26.4) C: 10 (9.4–22.1); spontaneous IPH hematoma volume at 24 hours post-reversal T: 9.2 (6.1–18.8), C: 9.9 (9.4–21.1); good outcome (mRS≤3) on discharge T: 10/28m C: 6/16; death/hospice on discharge T: 11/28 (39%), C: 6/16 (38%); length of hospital stay (median, IQR) T: 7 (4-15), C: 6 (2-11); length of ICU stay (median, IQR) T: 2 (1-4), C: 4 (1-8); thrombotic events T: 2/28, DVT 2 C: 0/16
Barra] et al., 2020. A retrospective single-center cohort study, US [18	Patients who received andexanet alfa or 4F-PCC for rivaroxaban- and apixaban-associated traumatic or spontaneous ICH N=29 (T=18, C=11); male: T=55.6%, C=81.8%; female: T=44.4%, C=18.2%; age (Median, IQR), T: 83.4 (70.3-88.8), C: 71.0 (68.6-73.2); GCS on admission (median, IQR), T: 15 (14-15), C: 10 (6-13); FXa inhibitor T: apixaban 15, rivaroxaban 3, C: apixaban 3, rivaroxaban 8	Andexanet alfa low-dose 400 mg IV bolus over 15 minutes followed by 480 mg infusion over two hours for last known apixaban or rivaroxaban dose ≥ 8 hours before administration, apixaban ≤ 5 mg with last dose < 8 hours prior or unknown, rivaroxaban ≤ 10 mg with last dose < 8 hours prior or unknown; high-dose: 800 mg IV bolus over 30 minutes followed by 960 mg infusion over two hours apixaban > 5 mg or unknown with last dose < 8 hours prior or unknown, rivaroxaban > 10 mg or unknown with last dose < 8 hours prior or unknown; low dose 18/18, high dose 0/18	C: 4F-PCC 25-50 units/kg, dosed per treating clinician discretion, with a maximum dose of 5000 units	Pre-reversal ICH volume T: 20.6 (2.0-41.3), C: 37.4 (22.6-88.2); post-reversal ICH volume T: 22.6 (2.0-51.7) C: 60.4 (33.2-106.7); hemostatic efficacy T: excellent 14/18, good 2/18, poor 2/18, C: excellent 6/10, poor 4/10 (one patient had no post-reversal imaging) Glasgow outcome score at discharge (median, IQR) T: 4 (3-4), C: 1 (1-3); in-hospital mortality T: 4/18 (22.2%), C: 7/11 (63.6%); ICU admission T: 14/18, C: 10/11; length of hospital stay (median, IQR) T: 6.3 (3.9-10.9); C: 4.7 (1.5-10.5); length of ICU stay (median, IQR); T: 2.7 (1.5-5.0) C: 2.1 (0.8-5.5); thrombotic events T: 3/18 DVT 2, superficial thrombosis 1, C: 1/11, superficial thrombosis 1
Brown et al., 2020. Retrospective multicenter case series, US [20]	Patients who received andexanet alfa for the reversal of factor Xa inhibitor-associated bleeding or reversal before surgical procedures N=25; male 10; female 15; age (median, IQR) 75 (71-83); indication for anticoagulation: Afib 15/25, DVT 9/25, peripheral arterial disease 1/25; FXa inhibitor: apixaban 20, rivaroxaban 5	Andexanet alfa low dose or high dose: low dose 19/25; high dose 6/25	None	ICH volume in cm3 at presentation (median, IQR) 40.3 (27.2-59.6); post-treatment hematoma volume in cm3 (median, IQR) 40.5 (20.45 – 47.95); mortality within 30 days 6/25 (24%); length of hospital stay (median, IQR) 4 (3-6); thrombotic events within 30 days 0/19
Coleman et al., 2020. A retrospective multicenter cohort study, US [19]	Patients who were hospitalized following major bleed due to FXai use N=3030 (T=342, C1=733, C2=925, C3=794, C4=438); male T=55%, C1=50%, C2=51%, C3=57%, C4=51%; female T=45%, C1=50%, C2=49%, C3=43%, C4=49%; age (mean) T: 69.1, C1:70.1, C2: 66.9, C3: 66.8, C4: 67.3; FXa inhibitor- T: apixaban 47%, rivaroxaban 50%, edoxaban 3%, C1: apixaban 51%, rivaroxaban 41%, edoxaban 8%, C2: apixaban 42%, rivaroxaban 52%, edoxaban 6%, others <1%, C3: apixaban 46%, rivaroxaban 49%, edoxaban 5% C4: apixaban 39%, rivaroxaban 56%, edoxaban 5%	T: Andexanet alfa	C1: 4F-PCC C2: FFP C3: Others (3-factor PCC, recombinant factor VIIa, activated 4F-PCC, tranexamic acid, and vitamin K) C4: No reversal administered	Inpatient mortality T: 12/342 (4%) C1: 74/733 (10%) C2: 105/925 (11%) C3: 67/794 (8%) C4: 34/438 (8%) length of hospital stay (median, IQR) T: 5.0 (3.0–6.0) C1: 5.0 (4.0–7.0) C2: 5.0 (4.0–8.0) C3: 5.0 (4.0–8.0) C4: 3.0 (1.8–5.0); length of ICU stay (median, IQR) T: 2.0 (1.0–4.0) C1: 3.0 (2.0–5.0) C2: 3.0 (2.0–5.0) C3: 3.0 (2.0–5.0) C4: 2.0 (1.0–3.0)
Connolly et al., 2019. Prospective multicenter, open-label, single-group study, North America and Europe [21]	Patients with acute major bleeding who had received within 18 hours one of the following: apixaban, rivaroxaban, or edoxaban at any dose or enoxaparin at a dose of at least 1 mg per kilogram of body weight per day. Exclusion criteria included planned surgery within 12 hours after andexanet alfa administration, ICH with GCS less than 7, hematoma volume more than 60 cc, expected survival less than one month, use of VKA, dabigatran, PCC, WB, or plasma in last seven days. Safety population: N1=352, male 187(53%); female 165(47%); efficacy population, N2=254, male 129(51%), female 125(49%), age (mean ± SD); safety population: 77.4 ±10.8; efficacy population: 77.1±11.1; indication for anticoagulation in the safety population; Afib 280/352, VTE 61/352, others 11/352; FXa inhibitor safety population: apixaban 194, rivaroxaban 128, edoxaban 10, enoxaparin 20; efficacy population: apixaban 134, rivaroxaban 100, edoxaban 4, enoxaparin 16	Andexanet alfa low dose or high dose: low dose, 400 mg IV bolus over 15 minutes followed by 480 mg infusion for all patients who had received apixaban and those who had received rivaroxaban more than seven hours before bolus administration. High dose, 800 mg iv bolus over 30 minutes followed by infusion 960 mg infusion for patients who had received enoxaparin, edoxaban, or rivaroxaban seven hours or less before bolus administration or at an unknown time. Low dose 208/249, high dose 41/249	None	Hemostatic efficacy 12 hours after the end of infusion: excellent 171/249, good 33/249, poor 45/249; percent change from baseline in anti-FXa activity after andexanet treatment (95% CI) at end of bolus: apixaban group: -92% (-93 to -91) rivaroxaban group: -92% (-94 to -88) enoxaparin group: -75% (-79 to -66); mortality within 30 days 49/352; thrombotic events within 30 days 34/352 MI 7, stroke 14, TIA 1, DVT 13, PE 5; a restart of any anticoagulation 220/352
Culbreth et al., 2018. Observational case series, US [23]	Patients with life-threatening bleeding who were on FXa inhibitor and received andexanet alfa: N=15, indication for anticoagulation; Afib 11/15; FXa inhibitor apixaban 8, rivaroxaban 7	Andexanet alfa low dose or high dose, low dose 11/15, high dose 4/15	None	Repeat CT scan: stable 8/14, worsening 6/14 (one patient died during surgery and didn’t have repeat CT); inpatient mortality 6/15 (40%); thrombotic events 0/15
Culbreth et al., 2019. Observational case series, US [22]	Patients who required emergent surgery after andexanet alfa administration for life-threatening bleeding: N=12; FXa inhibitor: apixaban 6, rivaroxaban 6	Andexanet alfa standard dose or high dose, standard dose 9/12, high dose 3/12	None	Hemostasis achieved as per surgeon 10/12, two required additional blood products; mortality at discharge 3/12; thrombotic events within seven days 0/12
Giovino et al., 2020. Retrospective case series, US [24]	Patients with spontaneous or traumatic ICH if were taking apixaban, rivaroxaban, or edoxaban and treated with andexanet alfa: N=39, male 24/39 (61.5%), female 15/39 (38.5%), age (Mean ± SD) 81.9 ± 9.3; Indication for anticoagulation: Afib 31/39, VTE 7/39, other 1/39; FXa inhibitor: apixaban 27, rivaroxaban 11, edoxaban 1	Andexanet alfa low dose or high dose, low dose 33/39 (84.6%), high dose 6/39 (15.4%)	None	Hemostatic efficacy on repeat CT excellent/good 29/35, poor 6/35; in-hospital mortality 4/39 (10.3%); length of hospital stay (mean ± SD) 5.4 ± 4.3; thrombotic events 1/39; bilateral pulmonary embolism
Nederpelt et al., 2020. Retrospective case series, US [25]	Patients (≥18 years old) who received andexanet alfa for the reversal of oral FXa inhibitor-associated extracranial hemorrhage, N=21, male: 13/21 (61.9%), female: 8/21 (38.1%), age (mean ± SD) 73.2 ± 15.4; indication for anticoagulation: Afib 16/21, recurrent popliteal thrombosis post-bypass 1, renal thrombosis 1, recurrent DVT 1, portal vein thrombosis 1, SVC occlusion 1; FXa inhibitor: apixaban 14, rivaroxaban 7	Andexanet alfa low dose or high dose, low dose 18/21 (85.7%), high dose 3/21 (14.3%)	None	Hemostatic efficacy: excellent 3/21, good 7/21, poor 11/2; in-hospital mortality 8/21 (38.1%), length of hospital stay (median, IQR) 9 (2.5-11), length of ICU stay (median, IQR) 2 (1.5-6.5), thrombotic events 4/21: stroke 2, PE 1, DVT 1, bowel ischemia 1, liver ischemia 1
Stevens et al., 2019. Retrospective case series, US [26]	Patients on oral FXa inhibitor with major bleeding who were prescribed andexanet alfa, N= 13, male: 7/13 (54%) female: 6/13 (46%), age (Mean ± SD) 69 ± 10; indication for anticoagulation: Afib 8/13, VTE 5/13; FXa inhibitor: apixaban 9, rivaroxaban 4	Andexanet alfa low dose or high dose based on FXa inhibitor type and dose and time of andexanet alfa initiation since the last dose of FXa inhibitor; low dose: 400 mg iv bolus followed by 480 mg IV infusion over two hours, high dose: 800 mg IV bolus followed by 960 mg IV infusion over two hours; low dose 11/13 (85%), high dose 2/13 (15%)	None	Hemostatic efficacy within 12 hours: excellent 8/13, good 2/13, poor 3/13; mortality within 30 days: 2/13 (15%); length of hospital stay (median, IQR) 14 (7-22); thrombotic events 4/13 MI 1, ischemic stroke 1, DVT 1, PE 1, superficial venous thrombosis 1, a restart of any anticoagulation 8/13

Quantitative Analysis

Only three studies reported the use of AA contrasting with 4F-PCC among ICH patient groups used in synthesis.

In-Hospital Mortality

**Figure 2:**
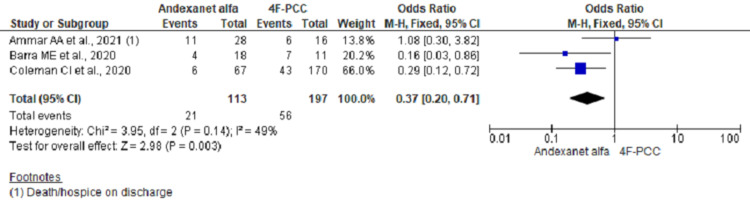
Forest plot showing mortality outcome using fixed effect model

**Figure 3:**
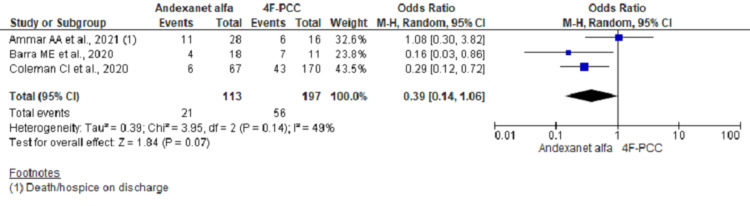
Forest plot showing mortality outcome using a random-effect model

**Figure 4:**
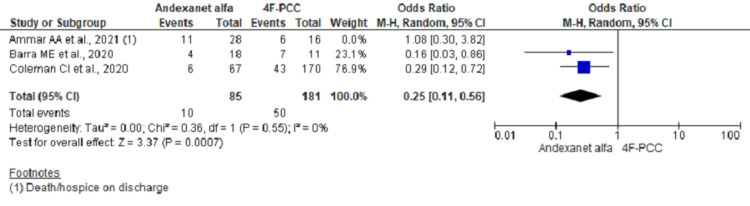
Forest plot showing mortality outcome using random-effect model (excluding Ammar AA et al.)

Pooling data on hospital mortality in ICH group using fixed effect model showed significant lower odds of mortality among AA group (OR, 0.37; 95% CI, 0.20-0.71; n= 310; I2 = 49%) (Figure 2). However, re-running the analysis using a random-effect model considering moderate heterogeneities across studies did not reach the statistical significance (OR, 0.39; 95% CI, 0.14-1.06) (Figure 3). Further analysis including two studies and excluding outlier study (Ammar et al.) showed significant lower odds of in-hospital mortality (OR, 0.25; 95% CI, 0.11-0.56; n = 266; I2 = 0%) (Figure 4).

Length of hospital stay

**Figure 5:**
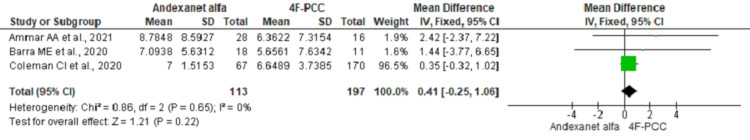
Forest plot showing the length of hospital stay outcome using fixed effect model

Length of stay in days did not differ significantly between treatment and control groups (MD, 0.41; 95% CI, -0.25 to 1.06; n = 310; I2 = 0%) (Figure 5).

ICU Length of Stay

**Figure 6:**
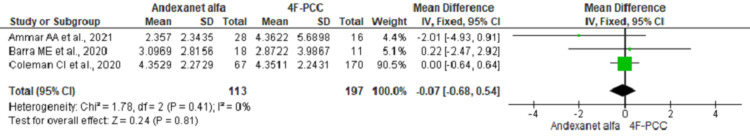
Forest plot showing the length of ICU stay outcome using fixed effect model

Length of ICU stay in days did not differ significantly between treatment and control groups (MD, -0.07; 95% CI, -0.68 to 0.54; n = 310; I2 = 0%) (Figure 6).

Thrombosis

**Figure 7:**
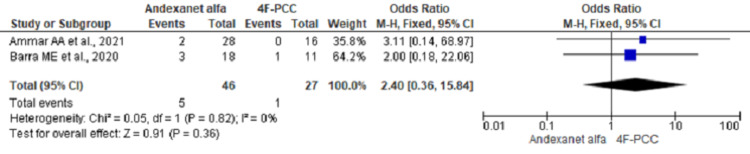
Forest plot showing thrombotic event outcome using fixed effect model

Thrombotic events were reported in two studies. Pooling of the data using fixed-effect model did not show significant differences between two groups (OR, 2.40; 95% CI, 0.36 to 15.84; n= 73; I2 = 0%) (Figure 7).

Discussion

Our meta-analysis is the most comprehensive meta-analysis to evaluate the effect of andexanet alfa in bleeding caused by factor Xa inhibitors evaluating the mortality, length of hospital stay, length of ICU stay, and thrombosis in comparison to 4-F PCC. The major finding of our study was that andexanet alfa decreased mortality in patients who had intracerebral bleeding due to factor Xa inhibitors compared to 4F-PCC. There were 105 mortalities in 865 patients (12.13%) receiving andexanet alfa across ten studies. In contrast, the overall mortality rate in a recent 4F-PCC meta-analysis in FXa inhibitor bleeding was 18% compared to 12.13% in our analysis and 14% in the ANNEXA-4 trial [27]. The studies done by Ammar et al. and Barra et al. showed a higher mortality rate of 39% and 22% respectively which is higher than that of other studies as these studies included only ICH patients [17,18]. Mortality was also significant in a study done by Culbreth (40%) as 14 out of 15 patients had ICH. The Ammar et al. study showed a similar mortality rate in the andexanet group and 4F-PCC group (39% and 38% respectively) while the Barra et al. study showed higher mortality in the 4F-PCC group (63.6%) than in andexanet receiving patients (22.2%) [17,18]. Patients in the 4F-PCC group in the Barra et al. study had lower baseline GCS and higher baseline hematoma volume which might have contributed to the higher mortality [18].

We found no difference in the incidence of thrombotic events caused by AA in comparison to 4F-PCC for the reversal of bleeding caused by factor Xa inhibitor. A recent meta-analysis of seven studies including 240 patients showed thrombotic events of 4% with the use of 4F-PCC [27]. In contrast, we found 48 incidences of thrombosis among 523 patients in the nine studies included in our analysis. A prior meta-analysis done by Rodrigues et al. had estimated the risk of thrombosis with andexanet alfa and Idarucizimab at 5.5%, however, the analysis just included three studies for evaluation of thrombosis risk associated with AA and evaluated the cumulative risk of thrombosis associated with both andexanet alfa and Idarucizimab [28]. The incidence of thrombotic events ranged from none to 30.7%, with a relatively higher incidence in studies by Steven et al. (30.7%) and Nederpelt et al. (19%) [25,26]. Culbreth et al. did studies also done by Brown et al. and two studies in 2018, and 2019 had zero incidences of thrombotic events [20,22,23]. A retrospective study done by Coleman et al. did not include the incidence of thrombotic events [19]. The most common thrombotic event reported was DVT; 19 out of 48 patients with thromboembolic events had DVT. Only the Connolly et al. study had more incidence of stroke (14) than DVT (13) [21]. Ammar et al. reported no thrombotic event in the 4F-PCC group, while one event was reported in the Barra et al. study, which is fewer than that reported in the AA group [17,18]. Restarting anticoagulation showed a significant decrease of thrombotic events in studies by Connolly et al. and Stevens et al. [21,26]. Only one patient (8%) in the Steven et al. study and eight patients (2%) in the Connolly et al. study developed thrombotic events after restarting anticoagulation [21,26]. Concomitant use of additional blood products - platelets, PRBCs, and FFP was common in multiple studies. However, the association between the use of additional products and thrombotic events could not be made. Time-frame for reporting thrombotic events also differed between studies. Studies done by Smith et al. and Tao et al. on 4F-PCC use in factor Xa inhibitor bleeding showed 0/31 (0%) and 1/43 (2.3%) thrombotic events [29,30].

The definition of hemostatic efficacy and time since AA administration for determining efficacy was different in between studies. Hemostatic efficacy was measured and reported as good/excellent or poor following criteria used by Sarode et al in five studies which include the ANNEXA-4 study. The Ammar et al. study used different values for hematoma expansion, the Culbreth et al. 2018 study reported repeat CT as stable or worsening while the Culbreth et al. 2019 study which included patients requiring emergent surgery reported hemostatic effectiveness as per surgeon [17,22,23]. Coleman et al. did not study hemostatic efficacy while Brown et al. evaluated hemostatic efficacy in ICH and surgery requiring patients as hematoma expansion if there was >20% increase in pre-treatment hematoma volume or hematoma diameter [19,20]. ANNEXA-4 trial and the Steven et al. study evaluated hemostatic efficacy by measuring anti-factor Xa activity, at end of 12 hours while 24 hours was used as the time frame in a study by Barra et al. and Nederpelt et al. [18,25]. Effective hemostasis (excellent and good) was achieved in 81.9% of patients in the ANNEXA-4 trial [18]. The Nederpelt et al. study showed lower efficacy of 47.6% while the Barra et al. study showed higher efficacy of 88.8% [18,25]. Different inclusion and exclusion criteria of patients, a wide range of definitions of hemostatic efficacy, and a time frame for judging led to the difference in hemostatic efficacy. Recent studies on the hemostatic efficacy of 4F-PCC have shown efficacy rates between 80% and 87% [30,31]. We found no difference in the length of hospital and ICU stay in patients receiving andexanet alfa in comparison to 4 F-PCC for reversal of bleeding caused by Factor Xa inhibitors. The median and IQR of the length of hospital stay varied from 4(3-6) in the Brown et al. study to 14(7-22) in the Steven et al. study [20,26]. Patients comparatively stayed in the hospital for longer in studies: Stevens et al., 14(7-22) days and Nederpelt et al., 9(2.5-11) days. Length of hospital stay was relatively longer in the AA group than the 4F-PCC group in studies by Ammar et al. and Barra et al., while it was similar in the Coleman et al. study. The median and interquartile range of the length of ICU stay ranged from 2(1-4) in the Ammar et al. study to 2.7(1.5-5.0) in the Barra et al. study. Length of ICU stay was longer in the AA group than the 4F-PCC group in the Barra et al. study, while it was shorter in the AA group in the Ammar et al. study [22].

Clinical benefit of AA use was observed in bleeding due to factor Xa inhibitors in our analysis; however, the cost of stocking AA in most hospitals might be prohibitive for the immediate use for reversible DOAC related life-threatening bleeding. The median projected cost of andexanet alfa was $22,120/patient compared to $5670/patient for 4F-PCC. 4F-PCC currently is more widely available and less expensive, but that may change if the cost for AA comes down in the future [32]. 4F-PCC and andexanet alfa have not been compared in a prospective randomized clinical trial, and results of such studies are needed to inform clinical practice in DOAC related bleeding events. There is an ongoing randomized, multicenter clinical trial evaluating the efficacy and safety of andexanet alfa versus the usual standard of care in patients with ICH anticoagulated with a DOAC, which may be completed in 2023 [33].

Limitations of the study

Most of the studies included were case series and retrospective observational studies. Only one prospective study, the ANNEXA-4 trial, was included. There were control groups in only three of our studies which were all retrospective. The sample size was less in our studies. Therefore, there was a moderate to high risk of bias in our studies. ANNEXA-4 trial had wide exclusion criteria: planned surgery within 12 hours after andexanet alfa administration, ICH with GCS less than 7, hematoma volume more than 60 cc, expected survival less than one month, use of VKA, dabigatran, PCC, WB, or plasma in last seven days. Giovino’s study also excluded patients with GCS less than 7 and hematoma volume >60 ml [24]. However, patients requiring surgical intervention, patients who received other blood products before AA administration, unknown time of the last factor Xa inhibitor dose, patients with low GCS and higher hematoma volume were included in other studies. In real clinical practice, patients with low GCS and expected mortality of less than one month required AA administration and were included in other studies. Knowledge about the administration of other blood products and time since the last factor Xa inhibitor was not feasible due to the retrospective nature of some studies and were thus included. Culbreth et al. 2019 included patients with bleeding due to factor Xa inhibitor who required emergent surgery.

## Conclusions

Andexanet alfa reduced in-hospital mortality in patients who had bleeding due to factor Xa inhibitors compared to 4F-PCC. There was no difference in thrombotic events, length of ICU, and hospital stay between andexanet alfa and 4F-PCC. Thus, AA is a promising therapeutic agent for the reversal of factor Xa-associated bleeding. However, the cost of stocking AA in most hospitals might be prohibitive for the immediate use for reversible of DOAC related life-threatening bleeding. 4F-PCC currently is more widely available and less expensive, but that may change when the cost for AA decreases. More studies are required in the future to determine the effect of AA as compared to 4F-PCC in patients with DOAC-related bleeding other than intracranial bleeding.

## Appendices

Supplementary Material 1. Details of the search strategy

PubMed

("andexanet alfa" OR "andexanet" OR "andexanet alpha") AND "bleeding" AND ("Factor Xa inhibitor" OR "Factor Xa inhibitors")

Hits: 117

https://pubmed.ncbi.nlm.nih.gov/?term=%28%22andexanet+alfa%22+OR+%22andexanet%22+OR+%22andexanet+alpha%22%29+AND+%22bleeding%22+AND+%28%22Factor+Xa+inhibitor%22+OR+%22Factor+Xa+inhibitors%22%29

PubMed Central

("andexanet alfa" OR "andexanet" OR "andexanet alpha") AND "bleeding" AND ("Factor Xa inhibitor" OR "Factor Xa inhibitors")

Hits: 452

https://www.ncbi.nlm.nih.gov/pmc/?term=(%22andexanet+alfa%22+OR+%22andexanet%22+OR+%22andexanet+alpha%22)+AND+%22bleeding%22+AND+(%22Factor+Xa+inhibitor%22+OR+%22Factor+Xa+inhibitors%22)

Scopus

("andexanet alfa" OR "andexanet" OR "andexanet alpha") AND "bleeding" AND ("Factor Xa inhibitor" OR "Factor Xa inhibitors")

Hits: 164

https://www.scopus.com/results/results.uri?src=s&st1=&st2=&sot=b&sdt=b&origin=searchbasic&rr=&sl=139&s=TITLE-ABS-KEY%20((%22andexanet%20alfa%22%20OR%20%22andexanet%22%20OR%20%22andexanet%20alpha%22)%20AND%20%22bleeding%22%20AND%20(%22Factor%20Xa%20inhibitor%22%20OR%20%22Factor%20Xa%20inhibitors%22))

Embase

Search: ('andexanet alfa'/exp OR 'andexanet alfa' OR 'andexanet' OR 'andexanet alpha'/exp OR 'andexanet alpha') AND ('bleeding'/exp OR 'bleeding') AND ('factor xa inhibitor'/exp OR 'factor xa inhibitor' OR 'factor xa inhibitors'/exp OR 'factor xa inhibitors')

Hits: 504

Link: https://www.embase.com/#advancedSearch/resultspage/history.1/page.1/25.items/orderby.date/source.

Cochrane Library

Hits: 9

"andexanet alfa" OR "andexanet" OR "andexanet alpha" in All Text AND "bleeding" in All Text AND "Factor Xa inhibitor" OR "Factor Xa inhibitors" in Title Abstract Keyword

https://www.cochranelibrary.com/advanced-search?cookiesEnabled
